# The Empirical Analysis of Cigarette Tax Avoidance and Illicit Trade in Vietnam, 1998-2010

**DOI:** 10.1371/journal.pone.0087272

**Published:** 2014-01-29

**Authors:** Minh Thac Nguyen, Ryan Denniston, Hien Thi Thu Nguyen, Tuan Anh Hoang, Hana Ross, Anthony D. So

**Affiliations:** 1 Department of Economics, University of Illinois at Chicago, Chicago, Illinois, United States of America; 2 Program on Global Health and Technology Access, Sanford School of Public Policy, Duke University, Durham, North Carolina, United States of America; 3 Department of Economics, Vietnam University of Commerce, Hanoi, Vietnam; 4 International Tobacco Control Research, American Cancer Society, Atlanta, Georgia, United States of America; 5 Program on Global Health and Technology Access, Sanford School of Public Policy and Duke Global Health Institute, Duke University, Durham, North Carolina, United States of America; Johns Hopkins Bloomberg School of Public Health, United States of America

## Abstract

Illicit trade carries the potential to magnify existing tobacco-related health care costs through increased availability of untaxed and inexpensive cigarettes. What is known with respect to the magnitude of illicit trade for Vietnam is produced primarily by the industry, and methodologies are typically opaque. Independent assessment of the illicit cigarette trade in Vietnam is vital to tobacco control policy. This paper measures the magnitude of illicit cigarette trade for Vietnam between 1998 and 2010 using two methods, discrepancies between legitimate domestic cigarette sales and domestic tobacco consumption estimated from surveys, and trade discrepancies as recorded by Vietnam and trade partners. The results indicate that Vietnam likely experienced net smuggling in during the period studied. With the inclusion of adjustments for survey respondent under-reporting, inward illicit trade likely occurred in three of the four years for which surveys were available. Discrepancies in trade records indicate that the value of smuggled cigarettes into Vietnam ranges from $100 million to $300 million between 2000 and 2010 and that these cigarettes primarily originate in Singapore, Hong Kong, Macao, Malaysia, and Australia. Notable differences in trends over time exist between the two methods, but by comparison, the industry estimates consistently place the magnitude of illicit trade at the upper bounds of what this study shows. The unavailability of annual, survey-based estimates of consumption may obscure the true, annual trend over time. Second, as surveys changed over time, estimates relying on them may be inconsistent with one another. Finally, these two methods measure different components of illicit trade, specifically consumption of illicit cigarettes regardless of origin and smuggling of cigarettes into a particular market. However, absent a gold standard, comparisons of different approaches to illicit trade measurement serve efforts to refine and improve measurement approaches and estimates.

## Introduction

The World Health Organization (WHO) ranked tobacco use the leading preventable cause of death in the world [1]. With 15.3 million adult smokers, Vietnam is one of the fifteen developing countries, identified by the Bloomberg Global Initiative to Reduce Tobacco Use, that jointly account for two-thirds of the world's smokers [2]. WHO estimated that 40,000 Vietnamese people die annually due to smoking, and 10% of the current Vietnamese population will die prematurely from tobacco related diseases [3]. Smoking also carries serious consequences for the economy, particularly with respect to health care costs borne by the government. Public expenditures came to 2,304 billion Vietnamese dongs (VND), or 121.3 million American dollars (USD), in 2007 to treat only three of the 25 diseases most closely related to tobacco use—lung cancer, ischemic heart disease, and chronic obstructive pulmonary disease [4].

Illicit trade carries the potential to magnify tobacco-related costs and problems through the increased supply of inexpensive, untaxed cigarettes. In fact, this is the rationale advanced by the tobacco industry in order to oppose policy changes and tax increases. For example, the industry has argued for lifting restrictions on kiddie packs, which contain fewer cigarettes than regular packs, with the rationale that these smaller, less expensive packs attract consumers away from illicit products and back to the legal market. Moreover, after an excise tax increase in 2006, illicit cigarette volume increased to 600 million packs in 2006, 636 million packs in 2007, and 731 million packs in 2008 [5], compared with 495 million packs in 2005 and 300–400 million packs between 1998 and 2004, as noted by a Vietnam Tobacco Association report [6].

Price differences, particularly with the countries of Laos and Cambodia that border Vietnam, play a role in illicit trade [7]. However, efforts to evade and undermine Vietnam’s import ban by transnational tobacco companies has been the central driver of smuggling in the past [7], [8]. Industry documents reveal that Singapore was one well-known origin or intermediary of smuggled cigarettes within the region, in particular the SE 555 brand [7], [9]. Prior to 2007, the Vietnamese government banned all cigarette imports, and the Vietnam National Tobacco Corporation (VINATABA) effectively held a monopoly over all legal cigarette supply. In early 2007, the cigarette import ban was lifted, and VINATABA was the sole company licensed by the government to import cigarettes. Prior to the opening of Vietnam’s market, internal documents describe BAT’s incorporation of contraband into the company’s business plan [7]. Active facilitation of cigarette smuggling by British American Tobacco (BAT) was devised as a means not only to evade the import ban and gain market share, but also to gain advantage in joint venture negotiations. Despite the Vietnamese government’s interest in sourcing cigarettes through local manufacture under the state-owned tobacco company, the demand built up from these smuggled brands reinforced the perceived demand for foreign cigarettes.

Although BAT was licensed to form a joint venture with VINATABA to plant tobacco and produce cigarettes in Vietnam, it still cultivated a perception among consumers that cigarettes manufactured domestically were inferior to those originating abroad [7], [8], [9]. To minimize business lost to domestic manufacture, BAT carefully managed consumer prices for both domestically produced and smuggled cigarettes. Most importantly, smuggled cigarettes cost somewhat more for consumers than those produced locally to protect the profits that emerged from the perception of quality and desirability held by the smuggled product, but not so much more that local brand production would be permanently undermined by a perception of inferiority [7]. More recent assessments find that smuggled brands retain a price premium, even after the lift of the import ban [9], [10], [11]. This price premium, particularly following the lift of the import ban, provides evidence that tax levels are not the sole driver of illicit trade.

The Vietnamese government has taken a number of steps to combat illicit trade with varying degrees of success. Crackdowns in 1990 [12] and 2002 [6] temporarily reduced cigarette smuggling. The imposition of tax stamps in 1999 by Decision No. 175/1000/QD-TTg also met with some success, as it facilitated the identification of illicit packs and counterfeit cigarettes, and reduced tax evasion by domestic firms due to sales under-reporting. However, illicit trade returned, and illicit cigarettes continued to command a price premium.

The lift of the import ban in 2006 did not reduce the flow of illicit cigarettes. Foreign cigarettes were moved by the government from the list of banned goods to the list of goods subject to business restrictions, and VINATABA was licensed as the only cigarette importer. According to a senior market control official, this action led to a lower level of oversight over foreign manufactured cigarettes and lighter penalties for cigarette smugglers, which raised smugglers’ incentives to smuggle and sell illicit cigarettes in Vietnam. Amid an increase in illicit volume, the government reinstated the banned goods classification, but smuggled cigarette volume fell only slightly in response [13].

While the relative importance of prices compared with consumer perceptions is not known, prices are an important factor for most countries, and taxes are an important component of consumer prices. Taxes on tobacco products have changed several times, beginning with the introduction of the excise tax in 1990 as shown in Table 1. Before 2006, assessed taxes depended on whether the cigarette contained a filter and whether the cigarette was predominantly composed of domestic or foreign materials. Excise taxes fell in 1998, but 7 months later, a value added tax modestly raised the tax burden placed on tobacco. The tiered excise system was eliminated through a tax increase in 2006, and taxes were raised again in 2008. At 41% and 45% of retail price in 2006 and 2008 respectively, Vietnam’s taxation level lies in the middle of regional tobacco tax rates, well above the 20% to 25% of retail price assessed by neighboring Cambodia, but far below the 72% assessed by Brunei and below the 60% to 80% range recommended by the World Bank [14].

**Table 1:** Excise tax rates for cigarettes and tobacco products in Vietnam, 1996–2011 (percent).

	Excise Tax			VAT
Period	Non-filter	Filter, mainly from domestic materials	Filter, mainly from foreign material	
January 1996 – May 1998	32	52	70	
June 1998 – December 1998	25	45	65	
January 1999 – December 2005	25	45	65	10
January 2006 – December 2007	55	55	55	10
January 2008 – January 2011	65	65	65	10

Sources: Guindon et al 2010.

Accurate and timely measurement of illicit trade is a vital contribution to tobacco control policy. This study contributes to this effort by advancing two methods, first identified by the World Bank [15], to estimate the consumption of illicit cigarettes and cigarette smuggling into Vietnam. These methods rely on public data that tend to be easy to obtain, relatively inexpensive to implement, and are transparent and replicable. Most existing estimates of illicit trade in Vietnam are industry sponsored and rely on opaque or undisclosed methods, or on methods that are difficult to implement repeatedly over time. Moreover, the existence of a financial conflict of interest merits the generation of independent measures of illicit trade. Industry studies may overstate the illicit cigarette trade in order to discourage efforts to increase taxes on tobacco products. Low cost, transparent, and independent methods may serve as a benchmark for future studies and for the development and implementation of new methods that promote a more complete understanding of the magnitude of illicit trade and its relation to tax and policy changes.

## Methods

The first method estimates the consumption of illicit cigarettes by calculating the discrepancy between tax-paid sales of cigarettes and a survey-based estimate of consumption. Consumption of illicit cigarettes is indicated where the consumption estimate exceeds tax paid sales. National health surveys only provide estimates of consumption of tobacco for the years of 1998, 2002, 2006, and 2010. While this method captures the magnitude of illicit cigarette consumption, it cannot measure the relative importance of illicit production and smuggling as sources for illicit cigarettes. Moreover, this method cannot measure the magnitude of cigarettes smuggled into and out of Vietnam. Based on conversations with officials from the Department of Market Control and the Ministry of Industry and Trade, this paper assumes that illicit production can be assumed nonexistent for the study period. If illicit production does exist, it would not alter the estimate of illicit cigarettes consumed, but it would lower estimates of net cigarettes smuggled into Vietnam because the sum of domestic illicit production and cigarettes smuggled into Vietnam must equal consumption of illicit cigarettes. Tax paid sales are measured by the total numbers of cigarettes sold domestically less net exports. Tax paid sales for all four years and cigarette exports through 2008 were provided by the Ministry of Industry and Trade, which in turn received data from the Vietnam Tobacco Association. Legal cigarette imports were effectively banned through 2006, except for duty-free sales, and are assumed zero for each year the ban was in effect. For 2010, net exports were sourced from the United Nations Comtrade database.

Tobacco consumption was estimated from the Vietnam Living Standards Survey 1998 (VLSS 1998), the Vietnam Household Living Standards Survey 2006 (VHLSS 2006), the Vietnam National Health Survey 2002 (VNHS 2002), and the Global Adult Tobacco Survey Vietnam 2010 (GATS 2010). The first three surveys were designed and conducted by the Vietnam General Statistics Office, while the Global Adult Tobacco Survey 2010 was a component in the Global Tobacco Surveillance System designed by the United States’ Centers for Disease Control and Prevention. Annual national cigarette consumption is the product of the average number of cigarettes smoked each day per smoker by gender and age group, the numbers of smokers in each group, and the number of days in a year. Only the VNHS 2002 and the GATS 2010 were detailed enough to calculate average cigarettes smoked per day. The VNHS 2002 average is applied to 1998 and 2006.

Although all surveys contained occasional smokers, only VNHS 2002 and GATS 2010 captured smoking intensity by occasional smokers. However, since their contribution to the estimated cigarette consumption were insignificant, 0.36% in 2002 and 0.04% in 2010, consumption was removed by occasional smokers from this calculation. Finally, most of literature suggests survey respondents understate their consumption of tobacco. The rates range from 22% to 31% in the United States between 1974 and 1985, respectively [16], from 28% to 30% in New Zealand in the period of 1976 and 1981 [17], and from 25% to 35% in Italy in the period of 2001 and 2008 [18], where social acceptability of smoking is less than it is in many developing countries. Given the continued high prevalence of smoking in Vietnam, the social acceptability of smoking is assumed to be equal or less than those of industrialized countries thirty years ago, and therefore three magnitudes of under-reporting, 10, 20 and 30% were applied to sensitivity analysis [16].

The second method estimates net smuggling into Vietnam using trade discrepancies that are summed across all trading partners in a given year. These discrepancies are the differences between imports recorded by Vietnam and exports to Vietnam recorded by each trading partner. Where exports recorded by trading partners exceed imports recorded by Vietnam, inward smuggling is indicated. Causes of trade discrepancies are both unintentional, like shipments made near the end of the calendar year and received the following year or accidental misreporting; and intentional, like trade diversion, tax evasion, and smuggling [19], [20]. Both the relative importance of unintentional and intentional causes of discrepancies and the contribution of each intentional cause of discrepancies cannot be ascertained. However, persistent and large discrepancies suggest illegal conduct [21]. This method directly estimates cigarettes smuggled into Vietnam. Illicit cigarette consumption will approximate cigarette smuggling if illicit production is not substantial, but will underestimate illicit cigarette consumption where illicit production is high. Trade data are sourced from the United Nations’ Comtrade database for 2000–2010. Cigarettes are classified by the commodity code Harmonized System (HS) 240220, cigarettes containing tobacco. Because both Vietnam and its trading partners did not record trade volumes consistently throughout the period, the results report value-based discrepancies. To compare trade discrepancies to the size of the domestic market, domestic cigarette revenues were converted to US dollars when available. The size of the domestic market as measured by value is only available for 2002 through 2006, and as value and volume measures may differ slightly due to unit value changes, this comparison will provide a general picture of the magnitude of smuggling in the context of market size.

## Results

Illicit cigarette consumption as measured by the first method above is present for at least some of the years for which surveys were available. Table 2 compares tax paid sales to the estimates of consumption with the several survey under-reporting scenarios. When no under-reporting by respondents is assumed, legal sales exceed consumption for each survey year. An assumption of between 10 and 30% under-reporting produces illicit cigarette consumption in three years-1998, 2002, and 2006. For example, if respondent under-reporting is assumed to be 10%, consumption of illicit cigarettes comprises nearly 6% of total consumption, the sum of legal sales and illicit cigarettes, in 2002. There is an increase in illicit cigarette consumption between 1998 and 2002, a slight decline between 2002 and 2006, and a sharp decline through 2010. It is important to note that the 2010 result may be inconsistent with the other results, as a different survey was used to estimate consumption. At its peak in 2002, illicit consumption accounted for between 6% and 20% of legal sales, depending on the under-reporting threshold used.

**Table 2:** Legal Cigarette Sales, Estimated Consumption, and Illicit Consumption, 1998–2010 (millions of packs).

Measure	1998	2002	2006	2010
Estimated consumption	2,008.69	3,201.59	2,594.88	2,569.38
Legal sales	2,195.00	3,365.00	3,425.00	4,920.5
Net exports	0.00	54.37	624.00	542.51
Consumption as proportion of legal sales (ratio)	0.92	0.97	0.93	0.59
Illicit consumption (no underreporting)	–186.31	–109.04	–206.12	–1,808.61
Illicit consumption (10% underreporting)	14.56	211.12	53.37	–1,551.67
Illicit consumption (20% underreporting)	215.43	531.28	312.86	–1,294.73
Illicit consumption (30% underreporting)	416.30	851.44	572.34	–1,037.80
Illicit consumption as share of total consumption (10% underreporting,%)	0.66	5.90	1.53	–46.06
Illicit consumption as share of total consumption (20% underreporting,%)	8.94	13.64	8.37	–35.71
Illicit consumption as share of total consumption (30% underreporting, %)	15.94	20.19	14.32	–26.73
For the estimated consumption to be equal to the legal sale, the underreporting is (%)	9.28	3.41	7.94	70.39

Sources: Authors’ calculation from VLSS 1998, VNHS 2002, VHLSS 2006, GATS Vietnam 2010 and Vietnam Tobacco Association’s reports.

Illicit trade is also observed when measured by the second approach, which measures smuggling by the sum of trade discrepancies across all trading partners. Smuggling declined from $250 million in 2000 to $110 million in 2003 as indicated by Table 3. However, smuggling rose to $300 million by 2010. Importantly, imports are small for all years, and compared to smuggled cigarettes, comprise less than 5% of all cigarettes entering Vietnam as reported by trading partners. This suggests that irrespective of changes in the magnitude of smuggling over time, smuggling itself remains a substantial problem.

**Table 3:** Net Smuggling into Vietnam and Its Share of Domestic Consumption (millions of US dollars).

Year	Imports by Vietnam	Exports to Vietnam	Smuggling into Vietnam	Smuggling as Share of Total Trade	Domestic Revenue	Smuggling as Share of Domestic Consumption
2000	0.4	258.8	258.4	99.7%	NA	NA
2001	0.7	271.6	270.9	99.5%	NA	NA
2002	1.3	187.5	186.2	98.6%	696.9	21.1%
2003	2.3	112.5	110.2	96.0%	853.4	11.4%
2004	3.6	158.9	155.3	95.6%	1009.9	13.3%
2005	3.4	158.8	155.4	95.8%	1153.1	11.8%
2006	2.7	195.2	192.5	97.3%	1160.3	14.2%
2007	2.5	196.3	193.8	97.5%	NA	NA
2008	4.2	264.8	260.6	96.9%	NA	NA
2009	1.6	287.3	285.7	98.9%	NA	NA
2010	0.1	305.3	305.2	99.9%	NA	NA

Sources: United Nations Comtrade Database, Vietnam Tobacco Association 2008.

Smuggling accounted for 11 to 21 percent of domestic consumption, the sum of legal domestic cigarette revenues and the value of smuggled products, between 2002 and 2006, the only years for which domestic revenues were available in US dollars. These estimates are somewhat larger than those provided by the first method, which compares tax paid sales to survey-based estimates of consumption. Finally, Singapore and Hong Kong account for nearly 80% of smuggled cigarettes as shown in Table 4. As a proportion of smuggling, the share held by Singapore and Hong Kong generally fell through 2008 and rose slightly after. By contrast, the importance of other regional partners rose over the decade. As of 2010, both Australia and Malaysia were the origin of roughly 5% of cigarettes smuggled into Vietnam, and Malaysia exceeded 10% in 2008.

**Table 4:** Net Smuggling into Vietnam among Ten Largest Sources for Smuggling, 2000–2010 (millions of US dollars).

Partner	2000	2002	2004	2006	2008	2010	Share of Total Discrepancy, 2000–10
Singapore	128.7	88.5	33.2	91.3	100.7	140.5	43.8%
Hong Kong SAR	115.5	83.2	93.7	41.9	69.6	100.4	35.5%
Macao SAR	9.2	9.4	8.2	14.7	18.0	0.0	5.1%
Malaysia	1.9	1.9	4.0	16.1	32.4	13.9	5.0%
Australia	0.0	0.3	0.2	9.2	14.0	16.2	2.9%
Philippines	0.6	0.3	14.4	9.5	9.6	4.1	2.7%
Indonesia	1.9	1.3	0.1	2.8	8.7	9.9	2.0%
China	0.0	0.3	1.5	4.2	1.8	2.7	1.0%
Thailand	0.3	0.7	0.1	0.5	1.1	5.5	0.6%
India	0.1	0.2	0.0	1.1	1.8	2.3	0.5%
Others	0.0	0.1	0.0	1.2	2.8	10.0	0.7%
World	258.4	186.2	155.4	192.5	260.6	305.2	

Source: United Nations Comtrade Database.

## Discussion

Both methods employed by this research indicate the presence of illicit trade in the early to mid 2000s. When illicit trade is measured by the consumption of illicit cigarettes using the first method outlined above, and when respondent under-reporting of consumption is assumed to be 10%, illicit cigarette consumption represents 0.7% of illicit and legal cigarette consumption in 1998, roughly 6% in 2002 and 1.5% in 2006. At 30% respondent under-reporting, the share of the domestic market composed of illicit cigarettes rises to 16% for 1998, 20.2% for 2002, and 14.3% for 2006. Under-reporting would have to be about 15% in 2002 and 30% in 2006 to produce estimates of illicit cigarette consumption that approximate those produced by the industry. These results provide a transparent and replicable benchmark for estimates produced by other methodologies, data sources, and periods.

When illicit trade is measured by smuggling using trade discrepancies, it is of greater magnitude than observed by the measure of illicit cigarette consumption when no under-reporting is assumed. However, this comparison is limited to a portion of the study period because value-based measures of the size of the domestic market were only available for 2002 through 2006. Smuggling constituted between 11.4% and 21.1% of the domestic market between 2002 and 2006. For the first method to achieve comparable estimates, respondent under-reporting must be assumed to be roughly 30%. The 30% threshold produces domestic market share estimates for illicit cigarettes of 14.3% and 20.2% respectively.

More importantly, the trends observed by each method diverge after 2006, with smuggling increasing but illicit cigarette consumption decreasing. A number of factors may account for this difference. First, the use of different data sources for tax paid sales, net exports, and a different survey to estimate legal consumption may produce an estimate of illicit cigarette consumption in 2010 that is inconsistent with those produced for other survey years. While legal sales rose by more than 40% between 2006 and 2010 alone, population rose by only 4% and cigarette consumption fell between 2006 and 2010. While net exports generally rose over the study period and could account for some of the increase in production, net exports also fell between 2006 and 2010. An apparent and growing surplus of cigarettes in the Vietnamese market suggests that it is possible that the estimates of consumption, legal sales, or both are inaccurate for 2010.

It is also important to note that the two methods measure illicit trade in different ways, and neither comprehensively captures the concept. The first method measures the magnitude of illicit cigarettes present in the market, but cannot distinguish whether these cigarettes originated domestically or abroad, and cannot measure the extent to which a country may also be a large source for illicit cigarettes for other countries. By contrast, illicit trade as measured by smuggling can only measure international flows of illicit cigarettes, not the possible presence of illicit cigarette production within a country. Consequently, the assumption that illicit production does not exist in Vietnam may not be valid, and Vietnam may be both a large recipient of and origin for smuggled cigarettes.

Because survey-based measures of consumption are not available on an annual basis, estimates based on these surveys are coarser than estimates of cigarette smuggling produced with trade discrepancies. This reduces the comparability of the two data series, as short-term trends and annual fluctuations are obscured. Were annual data available, they may produce estimates that align more closely with the estimates observed by the trade data. Finally, a real fall in the consumption of illicit cigarettes may be present after 2006. In this event, undetected, outward smuggling to other countries would have had to increase.

Research on global illicit cigarette trade estimates that smuggled cigarettes account for 11.8% of the domestic market for middle income countries and 16.8% for low income countries on average, and only 9.8% of the domestic market in high income countries [22]. The estimates for Vietnam fall within the ranges for low and middle income countries. With respect to estimates produced by the comparison of consumption to tax paid sales, the existing literature indicates that respondent under-reporting of smoking may be as high as 30%. Given that this research was conducted in countries where cigarette smoking might be considered less socially acceptable than for Vietnam given Vietnam’s status as a developing country and smoking prevalence, estimates that assume respondent under-reporting of 30% represent the upper bounds of reasonable illicit trade estimates. Finally, the study notes that over three-quarters of the trade discrepancy in cigarettes with Vietnam traces to Singapore and Hong Kong.

Future, robust efforts to measure the magnitude of illicit trade must address several limitations encountered in this research. First, changes in data sources within time series raise the possibility of inconsistent results and may limit the coverage of the research. Second, different methods may produce different results for a variety of reasons, including conceptual differences in the object of measurement and differences in accuracy across data sources. However, absent a gold standard, the need for continued work to triangulate on accurate measures of illicit trade is best filled through comparison of different approaches to illicit trade measurement. Finally, robust assessment of the relation between tax or other policy changes and subsequent changes to future illicit trade must contend with both limited time series data that is often encountered and the existence of numerous, closely timed policy changes that may complicate assessment the effects of each change and may not be independent events. Beyond longer time frames, the incorporation of multiple countries and the shift of the level of analysis to the tax or policy change, rather than the country, may address these issues.

Increased excise taxes that result in higher tobacco prices reduce tobacco consumption and can increase tax revenues. Moreover, smuggling into Vietnam almost entirely originates from within the region, and in particular, from Singapore and Hong Kong. Focused, cooperative efforts in partnership with these particular countries may be more effective in reducing tax evasion and tax avoidance than a halt to further excise tax increases. Implementing the FCTC protocol on illicit trade that calls for effective tracking and tracing system will be the key to addressing concerns of policy makers as they discuss the use of tobacco taxes as a public health tool. There is also the need for more frequent monitoring and consistent data collection as underscored by some of the difficulties faced during this research process. Beyond data collection and monitoring, determination of the relative impacts of non-tax policy changes compared with tax changes, such as illicit trade crackdowns and removal of import bans, would clarify both the importance of the multiple, closely timed policy changes observed in Vietnam and the importance of trade to public health. Finally, this research provides quantitative estimates of the magnitude of illicit trade independent from those produced by the industry. However, the methods employed do not directly capture the same concept, and data limitations reduce the comparability of these methods to one another and each method over time. Comparison to existing industry and independent estimates of illicit trade and implementation beyond Vietnam may highlight the conditions under which these two methods will differ and will help to further evaluate and refine their application.
